# Validation of Duplex Ultrasound Graft Surveillance in the Immediate Postoperative Period

**DOI:** 10.1055/s-0039-1688435

**Published:** 2019-07-22

**Authors:** Christos Argyriou, George S. Georgiadis

**Affiliations:** 1 Department of Vascular and Endovascular Surgery, University General Hospital of Evros, “Democritus” University of Thrace, Alexandroupolis, Greece

**Keywords:** graft, surveillance, duplex ultrasound, femoral–femoral bypass

## Abstract

A femoral crossover polytetrafluoroethylene graft was performed in a patient immediately, after a failed iliac endograft limb recanalization, performed 2 years after endovascular aortic aneurysm repair. Although graft patency was confirmed clinically by palpation of groin pulses as well as noninvasively by segmental pressure measurements and Doppler examination, in the immediate postoperative period, duplex ultrasound failed to show any blood flow inside the graft lumen until the fourth postoperative day. Subcutaneous air and air tapped within the wall structure of the graft are possible explanation for the failure to show flow.

A 7-mm femoral crossover polytetrafluoroethylene (PTFE) graft was performed after a failed endovascular attempt to recanalize a thrombosed endograft limb ( Fig. 1 ) in a patient who underwent endovascular aortic aneurysm repair (EVAR) 2 years ago. Although graft patency was confirmed clinically by palpation of groin pulses as well as noninvasively by segmental pressure measurements and Doppler examination, in the immediate postoperative period, duplex ultrasound failed to show any blood flow inside the graft lumen until the fourth postoperative day. Additionally, to prevent operator issues as a confounding factor, we compared the amplitude of the received signal on the graft to that of the native vessel, which was significantly lower on the graft.

**Fig. 1:**
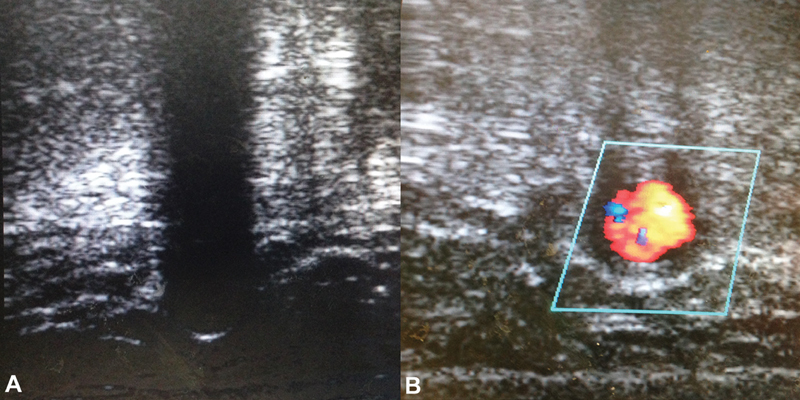
Duplex ultrasound showing the absence of blood flow inside the patent femoral–femoral graft lumen ( **A** ) until the fourth postoperative day ( **B** ).

Since the acoustic impedance and elastic stiffness of the expanded PTFE (ePTFE) graft wall are higher than in human native arteries, synthetic grafts greatly attenuate acoustical signal strength. 1 The cause for this attenuation is thought to be air trapped in the interstices of the ePTFE or between the fibers of Dacron. The ePTFE, which is used more frequently in femoral crossover bypasses, is a trilaminar structure with slight porosity and impermeability to liquid. Last but not least, a well-known plausible explanation for the failure to show graft flow is air trapped in the subcutaneous and subfascial planes intraoperatively, limiting full evaluation of the graft patency.

The phenomenon of reflective air within the interstices of an ePTFE graft, early on, merits attention of vascular experts, especially now that more and more femoral crossover bypasses are performed along with EVAR in patents with concomitant unilateral iliac artery chronic occlusion 2 or at secondary EVAR intervention after failed endovascular repair of an iliac endograft limb occlusion. 3

One decade long example of this phenomenon is, of course, the Viatorr tips endograft, which appears “occluded” early on, until the graft “wets out.” The Viatorr stent graft uses an ePTFE lining that is biocompatible, microporous, nonthrombogenic, and relatively impermeable to blood and tissue and provides a substrate for endothelial lining. The blood-contacting inner layer is made of ePTFE and has a microstructure and mechanical properties that are similar to those of the conventional vascular graft from the same manufacturer (GORE-TEX Vascular Graft; W.L. Gore, Flagstaff, AZ).

These observations may be helpful for the vascular surgeon's routine practice.
